# Influence of adding edible termite flour to *Ogi* powder: its chemical and phytochemical composition

**DOI:** 10.3389/fnut.2024.1403660

**Published:** 2024-07-05

**Authors:** Wasiu Awoyale, Funmilayo Racheal Fadeni, Busie Maziya-Dixon

**Affiliations:** ^1^Department of Food Science and Technology, Kwara State University, Malete, Kwara, Nigeria; ^2^International Institute of Tropical Agriculture, Ibadan, Oyo, Nigeria

**Keywords:** chemical composition, iron and zinc content, *Ogi* powder, phytochemical composition, supplementation, termite flour

## Abstract

*Ogi*, a traditional staple food made from submerged fermented cereal grains, is high in carbohydrates and low in protein. It is essential to conduct this research because termite flour (TF) addition may affect other quality aspects in addition to increasing protein content. Using 100 g of *Ogi* powder as a control sample, the chemical and phytochemical content of *Ogi* developed from blends of *Ogi* powder (OP) (50–100 g) with termite flour (TF) (10–50 g) was assessed using standard methods. The average proximate composition of the supplemented *Ogi* powder was 9.89% moisture, 3.87% fat, 2.59% crude fiber, 2.42% ash, 15.82% protein, and 65.41% total carbohydrates. Zinc is 3.19 mg/100 g while iron is 2.03 mg/100 g on average. Phytate (0.12 mg/100 g), oxalate (0.06 mg/100 g), saponin (0.73 mg/100 g), and tannin (0.02 mg/100 g) are phytochemical constituents. Though, supplemented *Ogi* powder of higher protein, ash, and iron contents than those of the control sample could be achieved by blending 50.0 g of OP with 50.0 g TF, 75.0 g of OP with 58.3 g TF, and 39.6 g OP with 30 g TF. However, blending 52.31% *Ogi* powder and 43.58% termite flour could produce a supplemented *Ogi* powder with nutritional and phytochemical constituents than those of the control sample. While the product could help lower the rate of protein-energy malnutrition, the supplemented *Ogi* powder’s amino acid, and carotenoid profiles need to be assessed.

## Introduction

*Ogi* is a fermented starchy paste traditionally made from maize, sorghum, or millet (1). It is considered the most important weaning food for infants in West Africa, although it is also consumed by older children and adults (2). Though the processing of cereal grain to *Ogi* is used to increase its shelf life, a significant loss of nutrients may occur via heat degradation or leaching, or several processing steps, which significantly reduce the amount of important nutrients like amino acids and minerals that are present, thus lowering the nutritional quality of the food (2). The nutritional value of *Ogi* is subsequently improved through the incorporation of additional nutrients from both plant and animal sources, such as pigeon pea, millet, cowpea, watermelon seed, African yam bean, egg, milk, and edible insects (3, 4).

The inclusion of edible insects in diets has the potential to improve global food and nutrition security. This is because consuming insects has been promoted as a healthy alternative to animal meat (5). In general, edible insects are abundant, rich in nutrients, and commercially valuable. Insects frequently have more protein, fat, and carbohydrate contents than equivalent amounts of beef or fish, as well as a higher calorie value than soybeans, maize, beef, fish, lentils, or other beans, according to research findings (6). Additionally, keeping insects results in a better conversion factor, expressed as kg of feed per kilogram of insect produced. In addition to being higher in protein and fat than the plants they feed on, edible insect caterpillars consist of comparable quantities and qualities of proteins, lipids, vitamins, minerals, and calories to those found in beef, fish, lamb, hog, chicken, milk, and eggs (5). Commonly consumed insects include adult crickets (*Brachytrypes* spp. and *Acheta domesticus*), adult short-horned grasshoppers (*Cytacanthacrisaeruginosus unicolor*), scarab beetle larvae (*Oryctesboas*), larvae of butterfly and moth (*Anaphe* spp.) and winged adult termites (*Macrotermesbellicosus/Macrotermesnotalensis*/*Tenebrio molitor*) (7). Besides beetles and grasshoppers, termites rank highest among the most consumed insect species in the world (7).

In many regions of the world, notably in Africa, termites (Isoptera), which are categorized as social insects with colonies, are commonly consumed. There have been 29 nations on three continents where termite use has been documented; Africa has the most reports, followed by the USA and Asia (7). In sub-Saharan Africa, Asia, Australia, and Latin America, termites have been consumed as food and are regarded as a delicacy that may be enjoyed as a meal or as a snack (8). Breastfeeding women have transformed dried termites into various unidentifiable like muffins and crackers and employed them as sprinkles in baby cereal (9). The major fat (44.82–47.31 g/100 g) and protein (33.51–39.74 g/100 g) contents of termites account for their significant status as edible insects in Africa (9).

Termites can provide food security in many developing and developed countries because they contain essential nutrients like protein, vitamins, minerals, and calories, which are often lacking in the diets of many people in these countries (5). Awoyale and Fadeni (10) worked on the influence of edible termite flour supplementation on the functional and pasting properties of maize *Ogi* powder and the sensory acceptability of the gruel. They found that an acceptable *Ogi* gruel of good pasting properties and comparable functionality to the 100% *Ogi* powder may be achieved by blending 75.0 g of *Ogi* powder with either 1.7 g or 30.0 g of termite flour. Still, no work has been reported on the chemical and phytochemical composition of *Ogi* powder supplemented with termite flour. Therefore, this study aimed to assess *Ogi* powder’s chemical and phytochemical composition supplemented with edible termite flour.

## Materials and methods

### Materials

The yellow maize grains were purchased from the Oja–Oba market in Nigeria’s Kwara State capital of Ilorin. Local anthills in the Malete community in Kwara State (8°42′0″ North, 4°28′0″ East) were used as a source of termites (Isoptera). A locally-made milling machine, a cabinet drier, muslin cloth, a sieve, plastic containers, and zip-lock bags were also used. These items were obtained from the Department of Food Science and Technology’s Food Processing Laboratory at Kwara State University in Malete, Kwara State.

### Methods

#### Preparation of *Ogi* powder

**Figure 1:**
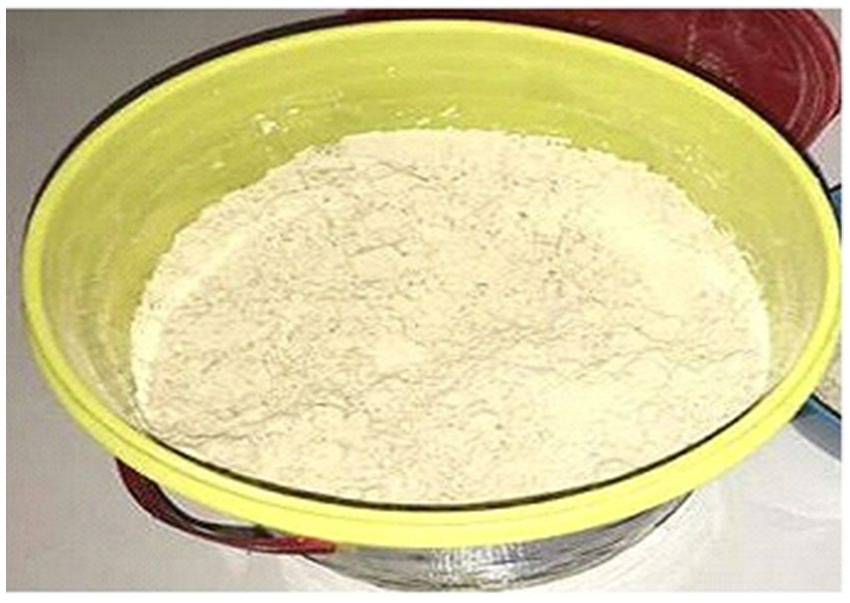
*Ogi* powder.

The wet-milling method outlined by Awoyale et al. (1) was used to produce *Ogi* powder (Figure 1). The yellow maize grains underwent cleaning, sorting, and a 48 h soaking in clean water at room temperature in a ratio of 1:3 (w/v) maize grains to water. The water was decanted, the fermented grains were rinsed in fresh water, and an attrition mill was used for wet milling. The filtrate was left to settle for 24 h to generate starchy sediment, which is *Ogi* slurry after the bran was removed moist using a muslin cloth. Using a hydraulic jack, the sediment was dewatered and placed in a jute bag. The dewatered mash was pulverized in a granulating machine, dried in a cabinet dryer (55 ± 5°C), and dry milled to pass a mesh sieve of 0.5 mm, then cooled down and packaged for further use.

#### Production of termite flour

**Figure 2:**
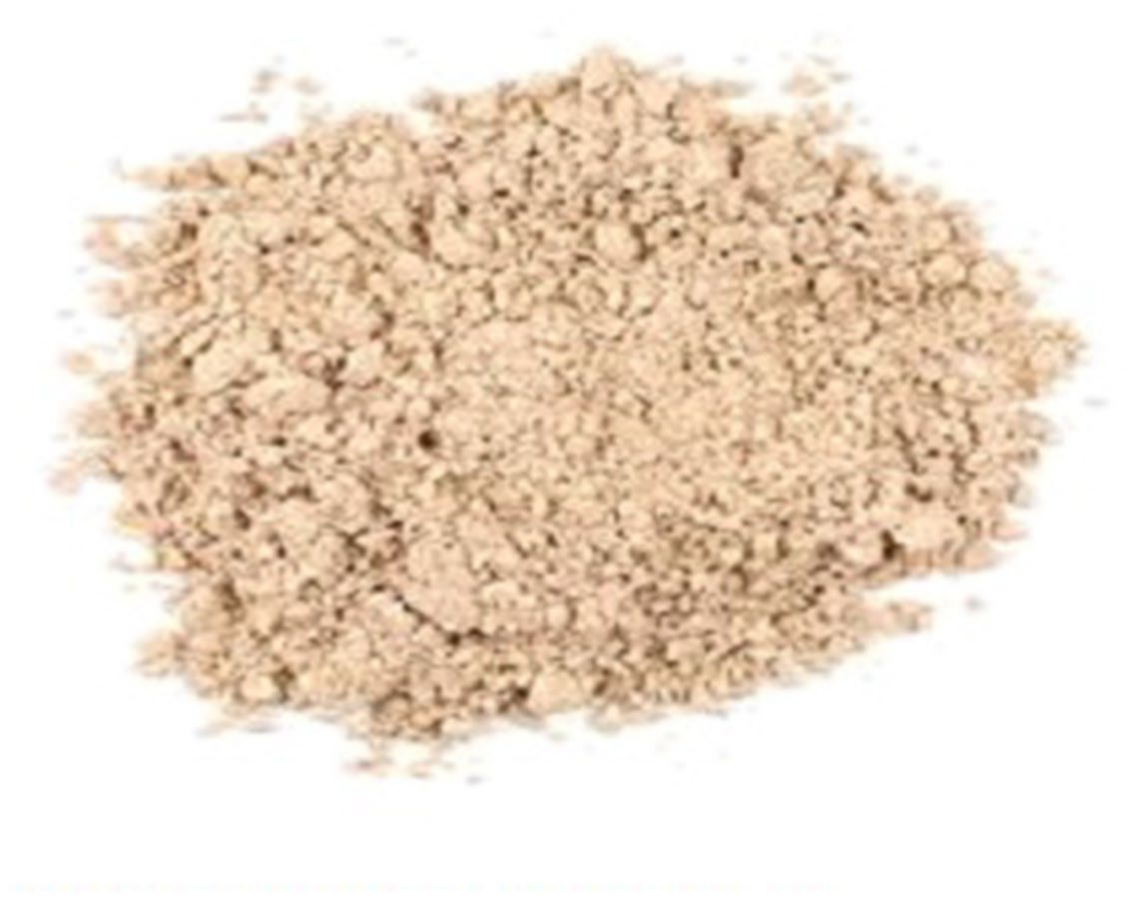
Termite flour.

Using the method described by Akullo et al. (11), fresh termites were picked into a basin of water to stop them from flying and then killed before turning them into flour. Before formulation, termites were cleaned by cutting off their wings and legs, then dried in an oven at 80°C for 10 min, roasted at 210°C for 15 min, ground in an attrition mill, cooled, and packed in a plastic container (Figure 2) before use.

#### Supplementation of *Ogi* powder with termite flour

Utilizing the ratios obtained from the Response Surface Central Composite Rotatable Design of the Design Expert Software (Version 6.0), the *Ogi* powder (100 g and 50 g) was supplemented with the termite flour (50 g and 10 g). This produced 13 runs with five central points (Table 1). Before doing additional investigations, the mixture was completely mixed in a laboratory blender, packaged, and sealed in a low-density polyethylene bag.

**Table 1:** Supplementation of *Ogi* powder with termite flour.

Runs	*Ogi* powder (g)	Termite flour (g)	Codes
1	75.0	30.0	SO1
2	75.0	1.7	SO2
3	75.0	58.3	SO3
4	75.0	30.0	SO1
5	75.0	30.0	SO1
6	110.0	30.0	SO4
7	75.0	30.0	SO1
8	100.0	50.0	SO5
9	50.0	10.0	SO6
10	75.0	30.0	SO1
11	100.0	10.0	SO7
12	39.6	30.0	SO8
13	50.0	50.0	SO9

#### Moisture content

The standard AOAC (12) official technique was used to evaluate the moisture content. About 1 g (*W*_1_) of the sample was dried in a hot air oven (Memmert GmbH+co.Kg, Oven model D-91126 Schwabach FRG, Germany) at 105 ± 1°C until constant weight (*W*_2_) was attained. The samples were then removed from the oven, cooled in a desiccator, and weighed. Equation (1) shows how the moisture content was expressed.


(1) $$\text{Moisture content}\;\left(\%\right)=\frac{W_{1}-W_{2}}{\text{Weight of sample}}\times 100$$

*W*_1_ = Mass of sample before drying (g)

*W*_2_ = Mass of sample after drying (g)

#### Ash content

A muffle furnace (Ney Vulvan TM furnace type 3-1750, USA) was used to measure ash content according to the established AOAC (12) method. Two grams (*W*_3_) of the sample were weighed into an ashing crucible that had already been weighed (*W*_2_), and the samples were then placed in the muffle furnace chambers at 700°C for 3 h, during which time they were reduced to ashes. After being taken out, the crucible was cooled in a desiccator and weighed (*W*_1_). Equation (2) shows how ash content was calculated as a proportion of the weight of the original sample.


(2) $$\text{Ash}\;\text{content}\;\left(\%\right)=\frac{W_{1}-W_{2}}{W_{3}}\times 100$$

*W*_1_ = Mass of crucible + Ash

*W*_2_ = Mass of empty crucible (g)

*W*_3_ = Weight of sample

#### Fat content

Using the Soxhlet equipment, the AOAC (12) method was employed to determine the amount of crude fat. A thimble containing around 5 g (*W*_3_) of the pulverized sample was put inside the Soxhlet extractor, and *n*-hexane was poured into a round bottom flask (*W*_2_) that had been pre-weighed to extract the oil from the sample. The extraction process took around 6 h to complete. Distillation was used to get the solvent out of the extracted oil. To get rid of any remaining organic solvent and moisture, the oil in the flask was given a second drying step in a hot-air oven at 90°C for 30 min. This was weighed (*W*_1_) after being cooled in a desiccator. Equation (3) was used to express the fat content as a percentage of the original sample.


(3) $$\text{Fat}\;\text{content}\;\left(\%\right)=\frac{W_{1}-W_{2}}{\text{Weight of sample}}\times 100$$

*W*_1_ = Weight of flask + oil

*W*_2_ = Weight of empty flask

#### Protein content

The Kjeldahl method was used to determine the protein content (12). The ground sample (0.20 g) was weighed into a Kjeldahl flask. One Kjeldahl tablet was added after 10 mL of pure sulfuric acid had been poured into the flask. To produce a clear solution, the mixture was digested on a heating racket. The digestate was cooled, made up to 75 mL with distilled water, transferred to a Kjeldahl distillation setup, and then 50 mL of a 40% sodium hydroxide solution was added. The ammonia that was formed in the mixture was then distilled into 25 mL of a 2% boric acid solution that contained 0.5 mL of a mixture of 100 mL of bromocresol green solution (prepared by dissolving 100 mg of bromocresol green in 100 mL of methanol) and 70 mL of methyl red solution (prepared by dissolving 100 mg of methyl red in 100 mL methanol) indicators. After that, 0.05 M HCl was titrated with the collected distillate. By leaving the sample out of the method, a blank determination was made. Equation (4) was used to calculate the protein content.


(4) $$\text{Protein content}\;\left(\%\right)=\frac{1.401\times M\times F\times \left(\text{mL}\;\text{titrant}-\text{mL}\;\text{blank}\right)}{\text{Weight of sample}}\times 100$$

*M* = Molarity of acid used (mol/dm)

*F* = Kjeldahl factor = 6.25

#### Crude fiber content

Using 2 g (*W*_3_) of the sample, the crude fiber was determined by AOAC’s (12) guidelines. The flask was filled with about 200 mL of 1.25% (v/v) sulfuric acid, put on a hot plate, and allowed to boil for 30 min. The filter paper was used to filter the content, and 50–70 mL of distilled water was used to remove any remaining residue. Removing the rinsed residue, 200 mL of 1.25% (w/v) NaOH was added, and the mixture was heated for 30 min. The content was then filtered as previously stated, and the residue that was left over was then cleaned with distilled water before being filtered once more with filter paper. After being moved to an ashing dish, the residue was dried at 130°C for 2 h, cooled in a desiccator, and weighed (*W*_1_). Then, for 30 min, it was ashed at 550°C in the muffle furnace (VULCANTM furnace type 3-1750) chamber, cooled and weighed again (*W*_2_). Equation (5) was used to calculate the crude fiber content.


(5) $$\text{Crude fiber content}\;\left(\%\right)=\frac{W_{1}-W_{2}}{W_{3}}\times 100$$

*W*_1_ = mass of crucible with dried residue (g)

*W*_2_ = Mass of crucible with the ash (g)

*W*_3_ = Weight of sample

#### Total carbohydrate content

Equation (6) illustrates how the carbohydrate content was calculated as a percentage of the difference between 100% and the addition of other proximate compositions.


(6) $$\text{Total carbohydrate}=100-\left(\begin{array}{l} \text{protein}+\text{fat}+\text{ash}\\ +\text{crude fiber}+\text{moisture} \end{array}\right)$$

#### Energy value

The energy value was calculated using the mathematical expression reported by Ijarotimi et al. (13) as shown in Equation (7)


(7) $$\text{Energy value}=\left[\left(\%\text{Fat}\times 9\right)+\left(\%\text{Protein}\times 4\right)+\left(\%\text{Carbohydrate}\times 4\right)\right]$$

#### Mineral determination

According to AOAC (12), an Atomic Absorption Spectrophotometer (Buck Scientific model 210 VGP) was used to determine the sample’s iron and zinc concentrations. Three hours at 600°C in a carbonite muffle furnace were used to incinerate 2 g of materials. Exact 10 mL of 6 N HCl were added to the ash sample, which was then put in a water bath and cooked for 10 min. The samples were taken out and put into a volumetric flask of 100 mL. Deionized water was used to dilute the filter paper until it was 100 mL in volume. The digested sample was divided into 10 mL, placed in a sample container, and aspirated into an atomic absorption spectrophotometer, and the amount of iron and zinc in parts per million (ppm) was recorded.

### Phytochemical composition

#### Phytate content

Phytate was measured using the Harland and Oberleas (13) method. About 2.4% HCl was used to extract the phytate from 5 g of the sample. The extract was deposited on an ion exchange column after being combined with an EDTA/NaOH solution. To liberate phosphate, which was then quantified colourimetrically, phytate was eluted with a 0.7 M HCl solution and wet digested with a solution of concentrated H_2_SO_4_ (0.5 mL) and HNO_3_ (3 mL). The original sample’s phytate content was estimated as hexaphosphate equivalent.

#### Saponin content

The determination of saponins was done using Brunner’s (14) spectrophotometric method. Isobutyl alcohol was used to extract the sample (5 g) for 2 h, and the filtrate was then combined with magnesium carbonate to create a colorless solution. In a UV-Spectrophotometer (Cecil CE 2502) at a wavelength of 380 nm, red saponin stock solution, and 5% red FeCl_3_ were added to produce red blood.

#### Tannin content

The AOAC (12) method was used to determine the amount of tannin. After dispensing the sample (5 g) into 50 mL of distilled water, it was shaken. After standing at 28°C for 30 min, the mixture was filtered using Whatman filter paper No. 3. A volumetric flask with a capacity of 50 mL received the extract (2 mL). A separate volumetric flask was filled with 2 mL of distilled water and 2 mL of standard tannic solution (0.1 mg/mL tannic acid). To each flask, 2.5 mL of saturated sodium carbonate (Na_2_CO_3_) solution and 1 mL of the Folin-C reagent were added, and the volume was then increased to 50 mL and thoroughly mixed. The sample was filtered using filter paper after standing for 1.5 h, and the absorbance at 760 nm was measured compared to a reagent blank. Equation (8) was used to calculate the tannin content.


(8) $$\text{Tannin content}=\frac{\text{Standard concentration}\times \text{Sample absorbance}}{\text{Standard absorbance}\times \text{Weight of sample}}$$

#### Oxalate content

The quantity of oxalate was determined by using the permanganate titration method described by Onwuka (15). In 100 mL of distilled water, the sample (5 g) was suspended, and 5 mL of 6 molar HCl was added. The mixture was heated at 100°C for 1 h to digest it, then cooled and filtered. A dropwise addition of concentrated aqueous ammonia solution (NH_4_OH) was then added after adding two drops of methyl red indicator to modify the pH until it was between pH 4 and 4.5, at which point a light yellow colouration developed. In a water bath, the mixture was heated to 90°C, cooled, and filtered (to get rid of ferrous ion precipitates). Once more heated to 90°C, the filtrate was then added with constant steering to 10 mL of 5% CaCl_2_ solution. After allowing the mixture to cool and spend a whole night in the refrigerator (5°C), it was centrifuged at 3,000×*g* for 6 min. The precipitate was dissolved in 10 mL of 20% H_2_SO_4_ after decanting the supernatant. Distilled water was used to prepare the solution up to 100 mL, and it was titrated against 0.05 KMnO_4_ solutions to reach a light pink color that lasted for 30 s. The formula: 1 mL of 0.05 M KMnO_4_ solution = 0.00225 g oxalate gave the oxalate content. The oxalate content was calculated using Equation (9).


(9) $$\%\text{Oxalate}=\left(\frac{100\times \text{titre}\times 0.0025}{W\;}\right)\times 100$$

Where *W* = weight of the sample used.

### Statistical analysis

Using the Statistical Package for Social Sciences (SPSS version 21) software, the obtained data were subjected to Analysis of Variance (ANOVA), the Pearson correlation, and the means were separated with Duncan’s multiple range test (*p* < 0.05). XLSTAT free version 2023 was used to carry out the Principal Component Analysis (PCA).

## Results and discussion

### Chemical composition of *Ogi* powder supplemented with termite flour

The mean of the chemical composition of the supplemented *Ogi* powder is moisture 9.89%, fat 3.87%, crude fiber 2.59%, ash 2.42%, protein 15.82%, total carbohydrate 65.41%, and energy value 359.71 kcal/ 100 g. The 100 g *Ogi* powder had the highest moisture (9.89%) and total carbohydrate (77.82%) contents. The fat (12.05%), crude fiber (5.11%), ash (4.07%), and protein (28.06%) contents and energy value (402.53 kcal/100 g) were higher in the 100 g termite flour (Table 2).

**Table 2:** Chemical composition of *Ogi* powder supplemented with termite flour.

Sample	Moisture content (%)	Fat content (%)	Crude fiber content (%)	Ash content (%)	Protein content (%)	Total carbohydrate content (%)	Energy value (kcal/100 g)
SO1	8.62 ± 0.02h	2.16 ± 0.00g	2.24 ± 0.01f	2.50 ± 0.01f	16.06 ± 0.03f	68.41 ± 0.04f	357.32 ± 0.11d
SO2	10.42 ± 0.01e	2.12 ± 0.00h	1.97 ± 0.01j	0.83 ± 0.01k	6.84 ± 0.01j	75.54 ± 0.01b	357.69 ± 0.02d
SO3	11.21 ± 0.01c	4.54 ± 0.00d	3.02 ± 0.01d	2.88 ± 0.01e	21.04 ± 0.01c	57.31 ± 0.01 h	354.20 ± 0.15d–f
SO4	10.31 ± 0.01f	2.23 ± 0.00f	2.12 ± 0.01g	2.07 ± 0.01g	12.66 ± 0.02g	70.60 ± 0.04e	355.32 ± 2.58de
SO5	12.05 ± 0.04b	2.42 ± 0.02e	2.42 ± 0.01e	3.04 ± 0.02d	20.65 ± 0.00d	59.41 ± 0.01g	349.63 ± 8.77f
SO6	12.02 ± 0.01b	2.15 ± 0.01g	2.00 ± 0.00i	1.44 ± 0.01i	7.16 ± 0.01i	75.24 ± 0.01c	350.99 ± 2.41ef
SO7	10.62 ± 0.01d	2.22 ± 0.01f	2.03 ± 0.01h	1.67 ± 0.00h	11.27 ± 0.00h	72.19 ± 0.02d	353.83 ± 0.07d–f
SO8	10.64 ± 0.01d	7.63 ± 0.02c	3.14 ± 0.02c	3.24 ± 0.01c	20.16 ± 0.00e	55.19 ± 0.01i	370.09 ± 0.19c
SO9	10.25 ± 0.01g	9.64 ± 0.01b	4.02 ± 0.01b	3.67 ± 0.00b	22.23 ± 0.03b	50.21 ± 0.02j	376.44 ± 0.11b
100 g OP	12.42 ± 0.01a	2.22 ± 0.01f	1.85 ± 0.00k	0.96 ± 0.00j	7.00 ± 0.02j	77.82 ± 0.01a	350.15 ± 0.02ef
100 g TF	5.25 ± 0.01i	12.05 ± 0.03a	5.11 ± 0.00a	4.07 ± 0.00a	28.06 ± 0.01a	45.65 ± 0.26k	402.53 ± 0.13a
Mean	9.89	3.87	2.59	2.42	15.82	65.41	359.71
*p* level	***	***	***	***	***	***	***

OP, *Ogi* powder; TF, Termite flour; SO1-75 g OP: 30 g TF, SO2-75 g OP: 1.7 g TF, SO3-75 g OP: 58.3 g TF, SO4-110 g OP: 30 g TF, SO5-100 g OP: 50 g TF, SO6-50 g OP: 10 g TF, SO7-100 g OP: 10 g TF, SO8-39.6 g OP: 30 g TF, SO9-50 g OP: 50 g TF, ***p < 0.001.

Means with different letters within the same column are significantly different (*p* < 0.05).

According to Awoyale et al. (16), a product’s initial moisture level determines its stability throughout storage. The lower the original moisture content of the *Ogi* powder, the better. The moisture content of the supplemented *Ogi* powder ranged between 8.62 and 12.05%, with 75 g OP: 30 g TF (SO1) having the lowest and 100 g OP: 50 g TF (SO5) the highest (Table 2). As a result of the lower moisture level than the 10% required by the Codex Alimentarius Commission, supplemented *Ogi* powder from SO1 may have low water activity, which will reduce the growth of bacteria, yeast, and mould (17). However, to prevent moisture absorption, it is important to properly package and preserve the supplemented *Ogi* powder throughout marketing. The moisture content (8.53–9.79%) of *Ogi* powder from maize enriched with ginger and cinnamon reported by Emelike et al. (18) was within the range of values reported in this study. Conversely, the moisture content (4.14–5.38%) of the *Ogi* from yellow and white maize, finger millet, popcorn maize, and white and red sorghum reported by Ijarotimi et al. (19) was lower compared to that of this study. Also, lower moisture values (8.23–8.40%) were reported by Adepeju et al. (20) for *Ogi* -mango mesocarp flour blends, and Ezekiel et al. (21) for 100% white-seeded maize *Ogi*. The variation in moisture content may be attributed to differences in raw materials, food formulation, and processing techniques (22).

The fat content of the supplemented *Ogi* powder ranged between 2.12 and 9.64%, appearing higher in 50 g OP: 50 g TF (SO9) and lower in 75 g OP: 1.7 g TF (SO2) (Table 2). The fat content increased with an increase in termite flour inclusion which could be because of the high fat content of termites (12.05%). The fat content reported in this work was within the range of the recommended 10% for complementary foods (23). A similar range of fat content to that of this study was reported by Ijarotimi et al. (19) in *Ogi* from yellow and white maize, finger millet, popcorn maize, and white and red sorghum (4.96–10.76%), and Adepeju et al. (20) for *Ogi* -mango mesocarp flour blends (3.11–4.65%). Also, the fat content of complementary food enriched with termites (2.66–5.28%) reported by Adepoju and Ajayi (24), and Ezekiel et al. (21) for 100% white-seeded maize *Ogi* (3.67%) was within the values of this study.

The crude fiber content of the supplemented *Ogi* powder ranged between 1.97 and 4.02%, with SO9 having the highest and SO2 the least (Table 2). The inclusion of termite flour, which has been known to be high in dietary fiber (Chitin) may have contributed to the increase in crude fiber of supplemented *Ogi* powder (5). The high fiber content of the supplemented *Ogi* powder has several health benefits which include improving intestinal digestion and reducing constipation (5). The fiber content reported in this study was higher than the values reported in *Ogi* powder (0.20–0.40%) from yellow and white maize, finger millet, popcorn maize, and white and red sorghum (13), and *Ogi* powder from pro-vitamin A maize genotypes (0.37–0.85%) (25), but almost like the values (1.20–4.04%) reported by Anyiam et al. (26) in fermented cassava mahewu supplemented with termites, Adepeju et al. (20) for *Ogi* -mango mesocarp flour blends (2.40–3.49%), and Ezekiel et al. (21) for 100% white-seeded maize *Ogi* (2.06%). The variation in the crude fiber content could be because of the difference in raw materials used and processing methods.

Ash, a nutritional indicator of food’s mineral content that is also known to help with the metabolism of other organic compounds like fat and carbohydrates ranges in value between 0.83 and 3.67% in the supplemented *Ogi* powder with SO2 having the lowest value and SO9 the highest (Table 2). The inclusion of the termite flour may have contributed to the high ash content of the supplemented *Ogi* powder (24). The Codex Alimentarius guidelines (18) state that a food is considered high in ash if ash content is more than 1%, a requirement that the supplemented *Ogi* powder meets. The ash content (1.53–2.53) of plantain and breadfruit biscuits supplemented with termites reported by Ani et al. (27) was within the range of the values in this study. A similar range of values was also noted for *Ogi* -mango mesocarp flour blends (2.42–2.75%) (20) and *Ogi* powder from yellow and white maize, finger millet, popcorn maize, and white and red sorghum (0.93–0.99%) (13). Conversely, the ash contents of malted maize flour supplemented with termites (3.87%) were higher than the values reported in this study (28), which could be due to the difference in processing methods and the raw materials used. Also, the ash content of *Ogi* powder from the pro-vitamin A maize genotype (0.48–0.52%) was lower compared to that of this study (25). The variation in the ash content could be attributed to the difference in raw materials and processing methods.

The protein content of the supplemented *Ogi* powder ranged between 6.84 and 22.23%, with SO9 having the highest and SO2 the least (Table 2). Termites are a good source of protein as evidenced by the increase in protein content of the supplemented *Ogi* powder. The work of Ezeocha et al. (28) on termite-supplemented malted maize food (12.68%), Ijerotimi et al. (19) on *Ogi* powder from yellow and white maize, finger millet, popcorn maize, and white and red sorghum (9.70–12.85%), Adepeju et al. (20) on *Ogi* -mango mesocarp flour blends (12.42–13.77%), and Akinsola et al. (25) on *Ogi* powder from pro-vitamin A maize genotype (7.82–8.72%) were within the range of values of this investigation. Hence, the supplemented *Ogi* powder in the present study may be beneficial for the management of protein-energy malnutrition (19).

The total carbohydrate content of the supplemented *Ogi* powder ranged from 50.21% in SO9 to 75.54% in SO2 (Table 2). The carbohydrate content of the supplemented *Ogi* powder decreases with an increase in termite flour inclusion which could be attributed to the low carbohydrate content of the termite flour, and the submerged fermentation process involved in the *Ogi* production. This is consistent with findings from studies by Fadahunsi and Soremekun (29) and Boyiako et al. (30) which showed that starchy foods typically have fewer carbohydrates when they undergo fermentation. A similar range of values to that of this study was reported by Ijarotimi et al. (19) for *Ogi* from yellow and white maize, finger millet, popcorn maize, and white and red sorghum (72.63–75.86%), and Adepeju et al. (20) for *Ogi* -mango mesocarp flour blends (68.53–70.83%). However, higher carbohydrate content was reported for *Ogi* powder from the pro-vitamin A maize genotypes (77.47–79.51) compared to this study (25).

The energy value of the termite flour (402.53 kcal/100 g) was higher than that of the *Ogi* powder (350.15 kcal/100 g). However, among the blends, the SO9 (376.44 kcal/100 g) had the highest energy value and the SO5 (349.63 kcal/ 100 g) had the lowest (Table 2). The high energy value of the SO9-supplemented *Ogi* powder may be attributed to its high fat, protein, and carbohydrate contents (19). The energy value of yellow maize *Ogi* powder (428.03 kcal/100 g) and that of white maize (427.56 kcal/100 g) were higher than the energy value of the supplemented *Ogi* powder of this study (19). This may be attributed to different processing methods, raw materials used, and the composition of the raw materials. However, a similar range of values was reported for the energy values (356.99–371.05 kcal/100 g) of *Ogi* -mango mesocarp flour blends (20).

### Iron, zinc, and phytochemical constituent of *Ogi* powder supplemented with termite flour

It is well known that minerals perform crucial metabolic and physiological functions in the living system Iron and zinc deficiency are some health problems faced by many women of reproductive age and children in developing countries (31). The mean values of the iron and zinc content of the samples in this study are 2.03 mg/100 g and 3.19 mg/100 g, respectively (Table 3).

**Table 3:** Iron and zinc, and phytochemical constituent of *Ogi* powder supplemented with termite flour.

Sample	Fe (mg/100 g)	Zn (mg/100 g)	Phytate (mg/100 g)	Oxalate (mg/100 g)	Saponin (mg/100 g)	Tannin (mg/100 g)
SO1	2.05 ± 0.00e	3.11 ± 0.00e	0.13 ± 0.00e	0.06 ± 0.01c	0.83 ± 0.00g	0.03 ± 0.03a
SO2	1.85 ± 0.00i	3.01 ± 0.00g	0.21 ± 0.01b	0.06 ± 0.00d	1.03 ± 0.00b	0.03 ± 0.00a
SO3	1.99 ± 0.00f	3.13 ± 0.00d	0.08 ± 0.00g	0.03 ± 0.00f	0.87 ± 0.00f	0.02 ± 0.00a
SO4	1.91 ± 0.00g	3.06 ± 0.00f	0.07 ± 0.00h	0.11 ± 0.01a	0.68 ± 0.00h	0.02 ± 0.01a
SO5	2.06 ± 0.00d	3.11 ± 0.00e	0.09 ± 0.00f	0.03 ± 0.00f	1.01 ± 0.00d	0.02 ± 0.00a
SO6	1.87 ± 0.00h	3.06 ± 0.00f	0.20 ± 0.00c	0.06 ± 0.00d	1.02 ± 0.00c	0.02 ± 0.01a
SO7	1.68 ± 0.00j	2.89 ± 0.00h	0.19 ± 0.01d	0.10 ± 0.01b	0.98 ± 0.00e	0.03 ± 0.00a
SO8	2.08 ± 0.00c	3.11 ± 0.00e	0.05 ± 0.00i	0.02 ± 0.00g	0.06 ± 0.00i	0.02 ± 0.00a
SO9	2.43 ± 0.00b	3.21 ± 0.00c	0.04 ± 0.01j	0.02 ± 0.00g	0.05 ± 0.00j	0.01 ± 0.00a
100 g OP	1.43 ± 0.00k	3.85 ± 0.00a	0.24 ± 0.00a	0.04 ± 0.00e	1.12 ± 0.00a	0.03 ± 0.01a
100 g TF	2.90 ± 0.00a	3.51 ± 0.00b	0.02 ± 0.00k	0.02 ± 0.00g	0.02 ± 0.00k	0.01 ± 0.00a
Mean	2.03	3.19	0.12	0.06	0.73	0.02
*p* level	***	***	***	***	***	NS

OP, *Ogi* powder; TF, Termite flour; SO1-75 g OP: 30 g TF, SO2-75 g OP: 1.7 g TF, SO3-75 g OP: 58.3 g TF, SO4-110 g OP: 30 g TF, SO5-100 g OP: 50 g TF, SO6-50 g OP: 10 g TF, SO7-100 g OP: 10 g TF, SO8-39.6 g OP: 30 g TF, SO9-50 g OP: 50 g TF, ***p < 0.001, NS, Not significant.

Means with different letters within the same column are significantly different (*p* < 0.05).

Iron is essential for brain development, oxygen transfer, and the prevention of anemia particularly in children whose fast growth puts them at risk (24). The SO9-supplemented *Ogi* powder had the highest iron content (2.43 mg/100 g) while 100 g OP: 10 g TF (SO7) had the lowest iron (1.68 mg/100 g) (Table 3). The iron content of this study was less than what was reported by Adepoju and Ajayi (24) in complementary food enriched with termite (2.81 and 4.13 mg/100 g). Conversely, the iron content of this study was higher compared to the iron content (0.12–0.14 mg/100 g) reported for *Ogi* powder from yellow and white maize, finger millet, popcorn maize, and white and red sorghum (19). The recommended intake of iron per day reported by WHO/FAO (23) is 4–8 mg/100 g of iron for infants and toddlers, which is higher compared to that of this study. However, the consumption of *Ogi* with iron-rich foods may provide the recommended daily intake.

Zinc on the other hand is necessary for growth, cell replication, fertility, reproduction, and its role in hormone production (24). The SO9-supplemented *Ogi* powder had the highest zinc content (3.21 mg/100 g) and SO7 had the lowest (2.89 mg/100 g) (Table 3). The zinc content reported in this study is lower than some of the values (4.12 mg/100 g) reported by Adepoju and Ajayi (24) for complementary food enriched with termite. However, Ijarotimi et al. (19) reported a lower range of values (0.80–1.07 mg/100 g) of zinc for *Ogi* powder from yellow and white maize, finger millet, popcorn maize, and white and red sorghum compared to that of this study. This may be due to different raw materials, and processing methods. The SO9-supplemented *Ogi* powder may contribute to the zinc daily recommended intake because Hlongwane et al. (31) reported the zinc daily recommended intake to be between 3 mg/100 g and 14 mg/100 g.

Edible insects have been reported to contain phytochemicals like phytate, tannin, and oxalate (5). The phytochemicals analyzed in this study include phytate, saponin, oxalate, and tannin whose mean values are phytate 0.12 mg/100 g, oxalate 0.06 mg/100 g, saponin 0.73 mg/100 g, and tannin 0.02 mg/100 g (Table 3). The phytochemical level in the supplemented *Ogi* powder reduced significantly (*p* < 0.05) with an increase in termite flour inclusion (except tannin content). This implies that termite is generally low in phytochemicals and the consumption of the supplemented *Ogi* powder may not pose a threat to the health of the consumers as it may act more like antioxidants rather than anti-nutrients.

Phytate, though antinutritional, is also known to act as antioxidants. When phytate is consumed excessively and unprocessed, it can hinder mineral absorption which leads to its classification as an antinutrient. However, real-world human data do not show mineral deficiencies from phytate intake especially when it has been processed and of low level, instead, it is regarded as a nutraceutical with potential health benefits like antioxidant and lipid-lowering effects (32, 33). The phytate content of this study ranged between 0.04 mg/100 g and 0.21 mg/100 g, with SO2 having the highest and SO9 the least (Table 3). These values agree with the recommended value (<0.70 mg/g) reported by NIS (34) The phytate content of the *Ogi* powder in this study was within the range of values (0.04–0.69 mg/100 g) reported by Ijarotimi et al. (19) in *Ogi* powder from yellow and white maize, finger millet, popcorn maize, and white and red sorghum.

Although saponins are regarded as antinutrients, research has demonstrated that they have positive impacts on the body such as lowering cholesterol, preventing diabetes, and preventing cancer (35). The saponin content of the supplemented *Ogi* powder ranged between 0.05 mg/100 g and 1.03 mg/100 g with SO2 having the highest value and SO9 the least (Table 3). The saponin content reduced significantly (*p* < 0.05) with an increase in termite inclusion, which may be because of the low amount of saponin in the termite flour. The saponin content (0.007–0.008 mg/g) reported by Sengev et al. (36) for *Ogi* produced from maize, millet, and sorghum supplemented with crayfish was lower than that of this study. Ijarotimi et al. (19) reported a saponin content of 0.54–1.77 mg/100 g for *Ogi* powder from yellow and white maize, finger millet, popcorn maize, and white and red sorghum, which was higher compared to that of this study. This variation may be attributed to differences in origin and species of grains.

The oxalate content of the supplemented *Ogi* powder ranged between 0.02 mg/100 g and 0.11 mg/100 g with 110.0 g OP: 30 g TF (SO4) having the highest value and 39.6 g OP: 30 g TF (SO8) and SO9, the lowest (Table 3). The oxalate values (0.33–0.95 mg/100 g) reported by Gwer et al. (37) for weaning food produced from millet, soybeans, and moringa leaf flour, were higher than those of this study. Also, the oxalate content of *Ogi* powder (0.0018–0.0162 mg/ 100 g) from yellow and white maize, finger millet, popcorn maize, and white and red sorghum was lower than those of this study (19). This variation may be attributed to differences in processing methods and the type of cereal grains used.

Tannins are naturally occurring plant polyphenols, like phytate as they bind and precipitate protein interfering with its digestion and absorption when present in large amounts and unprocessed (3). The tannin values ranged from 0.01 to 0.03 mg/100 g, with no significant difference (*p* > 0.05). The SO9-supplemented *Ogi* powder had the lowest tannin content and the SO1, SO2, and 100 g OP: 10 g TF (SO7) were the highest (Table 3). The tannin content of 0.02–0.06 mg/100 g reported for *Ogi* powder from yellow and white maize, finger millet, popcorn maize, and white and red sorghum was somehow higher than those of this study (19). This variation may be attributed to differences in processing methods and the type of cereal grains used.

**Figure 3:**
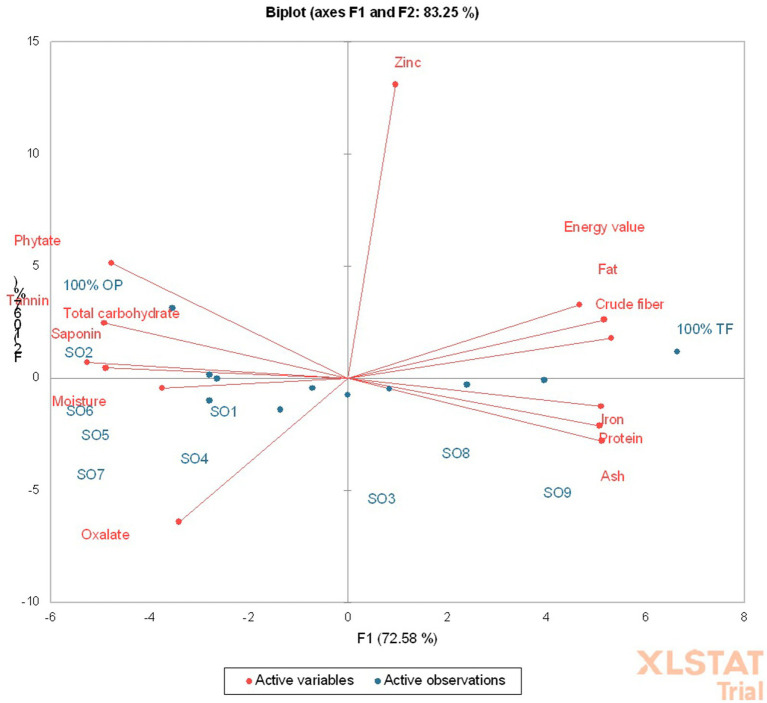
Principal component biplot of the chemical and phytochemical composition of termite supplemented *Ogi* powder.

The principal component analysis (PCA) depicts that a total of about 83.25% variation was observed, with the principal component (PC)-1 contributing 72.58% and PC-2 10.67% (Figure 3), meaning the PC-1 contributed the highest variance. The PC-1 had a positive correlation with fat (*p* < 0.05, *r* = 0.95), protein (*p* < 0.05, *r* = 0.93), ash (*p* < 0.05, *r* = 0.94), crude fiber (*p* < 0.05, *r* = 0.98), energy value (*p* < 0.05, *r* = 0.86), and iron (*p* < 0.05, *r* = 0.94) contents of the product (Table 4). Also, the PC-1 had a negative and significant correlation (*p* < 0.05) with the moisture (*r* = −0.69), total carbohydrate (*r* = −0.97), phytate (*r* = −0.88), oxalate (*r* = −0.63), saponin (*p* < 0.05, *r* = −0.90), and tannin (*r* = −0.90) contents of the product (Table 4). The 100 g OP and 75 g OP: 1.7 g TF-supplemented *Ogi* powder were in the same quadrant with the phytate, tannin, saponin, and total carbohydrate. The 100% termite flour was in the same quadrant with the energy value, fat, zinc, and crude fiber content. The SO8, 75 g OP: 58.3 g TF (SO3), and SO9-supplemented *Ogi* powder were in the same quadrant with the protein, ash, and iron contents. In addition, 50 g OP: 10 g TF (SO6), SO7, SO1, SO5, and SO4 were in the same quadrant with moisture and oxalate contents (Figure 3). Hence, supplemented *Ogi* powder of higher protein, ash, and iron contents than those of the control sample could be achieved from SO9, SO3, and SO8. However, comparable total carbohydrate content to that of the control sample may be produced from SO2.

**Table 4:** Correlation of the chemical and phytochemical composition of *Ogi* powder supplemented with termite flour.

Parameters	F1	F2
Moisture content	−0.69	−0.03
Fat content	0.95	0.18
Protein content	0.93	−0.15
Ash content	0.94	−0.20
Crude fiber content	0.98	0.13
Total carbohydrate	−0.97	0.05
Energy value	0.86	0.23
Iron content	0.94	−0.09
Zinc content	0.18	0.92
Phytate content	−0.88	0.36
Oxalate content	−0.63	−0.45
Saponin content	−0.90	0.03
Tannin content	−0.90	0.17

### The response surface methodology fitted models for the chemical properties of the termite flour supplemented *Ogi* powder

Predictive models for adjusting the amounts of *Ogi* powder and termite flour used in the formulations were established by fitting the experimental data to a common second-order polynomial equation for each response. To assess the significance of the linear, quadratic, and interaction effects of the independent factors on the dependent variables as well as the lack of fit (LoF), analysis of variance (ANOVA) was used (38). According to Awoyale et al. (38), the LoF test evaluates a model’s inability to adequately represent data in the experimental domain at points that were excluded from the regression and that the model can only be significant if the LoF is not significant. According to Awoyale et al. (38), the coefficient of determination (*R*^2^) is the percentage of response variable variability that the regression analysis accounts for. The empirical model more closely matches actual data when *R*^2^ is close to unity. The less relevance the dependent variables in the model have in explaining the variation’s behavior, the lower the *R*^2^ value (38). Awoyale et al. (38), also added that an *R*^2^ of at least 80% is required for a well-fitting model. For the model to be considered significant, the computed *F*-value needs to exceed the tabulated *F*-value, and the appropriate precision value needs to be higher than four (38).

Table 5 shows the response values, regression coefficient, *R*^2^, *F*-value, LoF, and adequate precision values of the chemical composition of the supplemented *Ogi* powder. The *R*^2^ for all the supplemented *Ogi* powder chemical properties was more than 80%, indicating significant models. The calculated *F*-values for all the chemical properties of the supplemented *Ogi* powder were more than the tabulated value (6.59–6.94), indicating the significance of the model for these properties. The LoF tests of all the supplemented *Ogi* powder responses were significant, suggesting the insignificance of the model for the chemical constituents. All the responses had adequate precision >4, indicating a significant model (Table 5). The effect of the independent variables (*Ogi* powder and termite flour) on the selected responses reveals that at a linear level, *Ogi* powder had a significant (except protein) negative effect (*p* < 0.05) on all the chemical constituents except total carbohydrate, which was positive and significant (*p* < 0.01). Similarly, termite flour had a significant positive effect on all the chemical constituents of the supplemented *Ogi* powder except moisture (*p* > 0.05) and total carbohydrates (*p* < 0.01). At the quadratic level, the *Ogi* powder had a significant positive effect on the moisture (*p* < 0.001), fat (*p* < 0.001), and crude fiber (*p* < 0.01), and a negative effect on the total carbohydrate content (*p* < 0.05). Also, termite flour had a significant positive effect on moisture (*p* < 0.001), fat (*p* < 0.05), crude fiber (*p* < 0.05), but a negative effect on the ash (*p* < 0.05), and total carbohydrate (*p* > 0.05). The interaction of *Ogi* powder and termite flour had a significant negative effect on the fat (*p* < 0.001), crude fiber (*p* < 0.001), and energy value (*p* < 0.001), but a significant positive effect on the moisture (*p* < 0.01), and total carbohydrate (*p* < 0.05) contents (Table 5). The *F*-value from the analysis of variance for all the responses, representing their total individual and combined effect at the linear, quadratic, and interactive level, showed that *Ogi* powder (except protein) and termite flour (except moisture) significantly (*p* < 0.05) affected all the chemical constituents positively at the total individual levels. The combined effect of the independent variables at the linear level was significant for all the chemical constituents except for moisture and energy values which were not significant (*p* > 0.05). At the quadratic level, all the chemical constituents were also significant except for protein and energy values, which were insignificant. At the combined interaction level, only fat, crude fiber, and energy values were significant (Table 5).

**Table 5:** Coefficient of second-order polynomial regression models and the analysis of variance results for the overall effect of process variables on the chemical composition of termite flour-supplemented *Ogi* powder.

Coefficient	^a^Moisture content (%)	^a^Fat content (%)	^b^Protein content (%)	^a^Ash content (%)	^a^Crude fiber content (%)	^a^Total carbohydrate content (%)	^c^Energy value (kcal/100 g)
*β*_0_	8.67	2.57	15.72	2.54	2.32	67.66	358.46
*β*_1_	−0.71*	−3.82***	−1.61	−0.49**	−0.73***	7.47**	−10.58***
*β*_2_	−0.11	2.45***	7.83***	1.21***	0.79***	−12.60***	4.66*
*β*_11_	2.74***	3.52***	NA	0.32	0.53**	−7.66*	NA
*β*_22_	2.46***	1.15*	NA	−0.53*	0.27*	−1.33	NA
*β*_12_	1.81**	−4.10***	NA	−0.49	−0.91***	6.90*	−22.14***
*R* ^2^	0.95	0.97	0.86	0.95	0.97	0.95	0.87
Adeq Precis.	12.10	20.03	16.34	19.16	23.26	17.44	16.79
*F*-value	25.28	42.69	31.79	28.25	49.61	25.18	20.11
LoF	S	S	S	S	S	S	S
Total individual effect (*F*-value of ANOVA)							
*Ogi* powder, *X*_1_	8.63*	116.7***	2.03	13.29**	85.88***	24.54**	27.83***
Termite flour, *X*_2_	0.31	68.26***	61.56***	115.61***	144.07***	98.83***	6.47*
Combined effect of all variables (*F*-value) at:							
Linear level	0.02	7.14*	31.79***	23.87***	13.02**	22.98***	3.73
Quadratic level	54.49***	21.77**	1.19	7.35*	11.80**	5.25*	1.38
Interactive level	1.31	7.16*	2.27	1.65	11.81**	3.21	30.72***
Interactive level	0.63		0.74	0.85	1.74	4.81	0.01

*p ≤ 0.05, **p ≤ 0.01, ***p ≤ 0.001, S, significant (*p* > 0.05), *β*_0_ = Regression coefficient for constant, *β*_1_ = Linear regression coefficient for *Ogi* powder (*X*_1_), *β*_2_ = Linear regression coefficient for termite flour (*X*_2_), *β*_11_ = Quadratic regression coefficient for *Ogi* powder, *β*_22_ = Quadratic regression coefficient for termite flour, *β*_12_ = Interactive regression coefficient for *Ogi* powder and termite flour, *R*^2^ = Coefficient of determinant, LoF = Lack of Fit, Adeq. Prec. = Adequate Precision, ^a^*F*-Tabulated = 6.94, ^b^*F*-Tabulated = 6.26, ^c^*F*-Tabulated = 6.59, NA = Not available.

Thus, the results of the chemical composition of the supplemented *Ogi* powder showed that the model for all the chemical constituent responses was highly adequate because they have satisfactory levels of *R*^2^ of more than 80%, calculated *F*-values were more than the tabulated value, and adequate precision was greater than 4 for all the responses (38). The negative effect of *Ogi* powder on the energy value of the supplemented *Ogi* powder may be due to the low level of protein and fat in its composition. The positive influence of termite flour on the energy value of the supplemented *Ogi* powder may be attributed to its high protein and fat content. The model for the chemical constituents of the supplemented *Ogi* powder could explain at least 86.41% of the variations in the chemical constituents. Hence, only 13.59% of the variation could be attributed to factors not included in the model. Consequently, the quadratic model may be suitable for predicting the moisture, fat, ash, crude fiber, and total carbohydrate contents of the supplemented *Ogi* powder as shown in Equations (10), (11), (13), (14), and (15), respectively. Conversely, the linear and two-factor interactive models may be used for predicting the protein and energy values of the supplemented *Ogi* powder as shown in Equations (12) and (16), respectively.


(10) $$\begin{array}{l} \text{Moisture content}=8.67-0.71\beta_{1}-0.11\beta_{2}\\ \;+2.74\beta_{11}+2.46\beta_{22}+1.81\beta_{12} \end{array}$$

(11) $$\begin{array}{l} \text{Fat}\;\text{content}=2.57-3.82\beta_{1}+2.45\beta_{2}+3.52\beta_{11}\\ \;+1.15\beta_{22}-4.10\beta_{12} \end{array}$$

(12) $$\text{Protein content}=15.72-1.61\beta_{1}+7.83\beta_{2}$$

(13) $$\begin{array}{l} \text{Ash}\;\text{content}=2.54-0.49\beta_{1}+1.21\beta_{2}\\ \;+0.32\beta_{11}-0.53\beta_{22}-0.49\beta_{12} \end{array}$$

(14) $$\begin{array}{l} \text{Crude fiber content}=2.32-0.73\beta_{1}+0.79\beta_{2}\\ \;+0.53\beta_{11}+0.27\beta_{22}-0.91\beta_{12} \end{array}$$

(15) $$\begin{array}{l} \text{Total carbohydrate content}=67.66+7.47\beta_{1}-12.60\beta_{2}\\ \;-7.66\beta_{11}-1.33\beta_{22}+6.90\beta_{12} \end{array}$$

(16) $$\text{Energy value}=358.46-10.58\beta_{1}+4.66\beta_{2}-22.14\beta_{12}$$

### The response surface methodology fitted models for the iron and zinc, and phytochemical composition of the termite flour supplemented *Ogi* powder

The response values, regression coefficient, *R*^2^, *F*-value, LoF, and adequate precision values of the iron and zinc, and phytochemical composition of the supplemented *Ogi* powder are shown in Table 6. Only phytate and saponin contents of the supplemented *Ogi* powder had an *R*^2^ of more than 80%, indicating significant models for these attributes. The calculated *F*-values for the iron and zinc, and all the phytochemical constituents of the supplemented *Ogi* powder were more than the tabulated value (6.26–6.94) except for tannin, indicating the significance of the model for these properties, except tannin. The LoF tests of all the supplemented *Ogi* powder responses were significant, except for the tannin content, which was not significant, suggesting the significance of the model for the tannin content. All the responses had adequate precision >4 except for the tannin content, which was lower. This indicates a significant model for iron and zinc, and all the phytochemical constituents except tannin content (Table 6). The effect of the independent variables (*Ogi* powder and termite flour) on the selected responses reveals that at a linear level, *Ogi* powder had a significant positive impact on the phytate (*p* < 0.05), oxalate (*p* < 0.01), and saponin (*p* < 0.001) content, but a significant and negative effect on iron (*p* < 0.05) and zinc (*p* < 0.05) contents. The linear effect of termite flour was positive and significant for iron (*p* < 0.01) and zinc (*p* < 0.001) contents, but negative and significant for phytate (*p* < 0.001), oxalate (*p* < 0.001), and saponin (*p* < 0.001) contents. At the quadratic level, the *Ogi* powder had a significant negative effect on the phytate (*p* < 0.01), and saponin (*p* < 0.01) contents. The interaction of *Ogi* powder and termite flour had a significant positive effect on only the saponin (*p* < 0.01) content (Table 6). The *F*-value showed that *Ogi* powder and termite flour significantly (*p* < 0.05) affected the iron and zinc, and all the phytochemical constituents positively at the total individual levels, except for tannin which was not significant. The combined effect of the independent variables at the linear level was significant for the iron and zinc, and all the phytochemical constituents except for tannin which was not significant (*p* > 0.05). It was only the phytate and saponin contents that were positively influenced (*p* < 0.01) at the quadratic level. The combined interaction between the *Ogi* powder and termite flour had no significant effect on the iron and zinc, and all the phytochemical constituents of the supplemented *Ogi* powder (Table 6).

**Table 6:** Coefficient of second-order polynomial regression models and the analysis of variance results for the overall effect of process variables on the iron and zinc, and phytochemical constituents of termite flour-supplemented *Ogi* powder.

Coefficient	^a^Iron content (mg/100 g)	^a^Zinc content (mg/100 g)	^b^Phytate content (mg/100 g)	^a^Oxalate content (mg/100 g)	^b^Saponin content (mg/100 g)	^a^Tannin content (mg/100 g)
*β*_0_	2.03	3.09	0.13	0.05	0.78	0.02
*β*_1_	−0.16*	−0.07*	0.03*	0.04**	0.48***	0.01
*β*_2_	0.20**	0.10***	−0.08***	−0.03*	−0.28**	−0.01
*β*_11_	NA	NA	−0.08**	NA	−0.52**	NA
*β*_22_	NA	NA	0.02	NA	0.17	NA
*β*_12_	NA	NA	0.03	NA	0.56**	NA
*R* ^2^	0.67	0.75	0.94	0.71	0.93	0.28
Adeq Precis.	9.28	11.22	16.50	10.25	14.81	3.95
*F*-value	10.25	15.31	21.46	12.27	19.47	1.95
LoF	S	S	S	S	S	NS
Total individual effect (*F*-value of ANOVA)						
*Ogi* powder, *X*_1_	6.83*	8.63*	9.46*	14.77**	50.54***	0.91
Termite flour, *X*_2_	13.66**	21.99***	79.96***	9.77*	23.69**	2.99
Combined effect of all variables (*F*-value) at:						
Linear level	10.25**	15.31***	11.24**	12.27**	4.06*	1.95
Quadratic level	0.76	1.71	12.58**	2.24	15.27**	0.42
Interactive level	0.63	0.74	0.85	1.74	4.81	0.01

*p ≤ 0.05, **p ≤ 0.01, ***p ≤ 0.001, S, Significant, NS, not significant (*p* > 0.05), *β*_0_ = Regression coefficient for constant, *β*_1_ = Linear regression coefficient for *Ogi* powder (*X*_1_), *β*_2_ = Linear regression coefficient for termite flour (*X*_2_), *β*_11_ = Quadratic regression coefficient for *Ogi* powder, *β*_22_ = Quadratic regression coefficient for termite flour, *β*_12_ = Interactive regression coefficient for *Ogi* powder and termite flour, *R*^2^ = Coefficient of determinant, LoF = Lack of Fit, Adeq. Prec. = Adequate Precision, ^a^*F*-Tabulated = 6.94, ^b^*F*-Tabulated = 6.26, NA = Not available.

Consequently, the model for phytate and saponin responses was highly adequate because they have satisfactory levels of *R*^2^ of more than 80%, calculated *F*-values were more than the tabulated value, and adequate precision was greater than 4 for all the responses (38). Based on the importance of the iron and zinc contents in the supplemented *Ogi* powder, and the satisfaction of other fit model conditions, the models for iron and zinc were considered notwithstanding their *R*^2^ of less than 80%. The positive effect of *Ogi* powder on the zinc, phytate, and saponin constituents of the supplemented *Ogi* powder may be due to the high level of these constituents in its composition, compared to termite flour. The positive influence of termite flour on the iron content of the supplemented *Ogi* powder may be attributed to the high iron content. The model for the phytate and saponin contents of the supplemented *Ogi* powder could explain at least 93.29% of the variations in the phytate and saponin. Hence, only 6.71% of the variation could be attributed to factors not included in the model. The model for iron and zinc could explain 67.20 and 75.38% of the variations respectively, while only 32.80 and 24.62% of the variation could be attributed to factors not included in the model for iron and zinc, respectively. Thus, the linear model may be suitable for predicting the iron and zinc contents of the supplemented *Ogi* powder as shown in Equations (17) and (18), respectively. The phytate and saponin content of the supplemented *Ogi* powder could be predicted using the quadratic model as shown in Equations (19) and (20), respectively.


(17) $$\text{Iron content}=2.03-0.16\beta_{1}+0.20\beta_{2}$$

(18) $$\text{Zinc content}=3.09-0.07\beta_{1}+0.10\beta_{2}$$

(19) $$\begin{array}{l} \text{Phytate content}=0.13+0.03\beta_{1}-0.08\beta_{2}\\ \;-0.08\beta_{11}+0.02\beta_{22}+0.03\beta_{12} \end{array}$$

(20) $$\begin{array}{l} \text{Saponin content}=0.78+0.48\beta_{1}-0.28\beta_{2}-0.52\beta_{11}\\ \;+0.17\beta_{22}+0.56\beta_{12} \end{array}$$

### Optimization of the variables regarding the modeled chemical and phytochemical constituents of the termite flour supplemented *Ogi* powder

The modeled chemical and phytochemical constituents as shown in Supplementary Figures 1, 2 were useful for indicating the directions where the independent variables were to be changed to maximize the attributes to produce the supplemented *Ogi* powder. These responses were used for the numerical optimisation of the independent variables, and the criteria used are presented in Table 7. Consequently, to produce a termite flour supplemented *Ogi* powder, which may contribute to the nutritional and phytochemical constituents, the response values of fat content, protein content, ash content, crude fiber, carbohydrate, energy value, iron, and zinc contents were maximally optimized while minimizing the moisture content. Also, phytate and saponin contents were kept within range as the phytochemicals. The *Ogi* powder and termite flour were also kept within range as the independent variables while optimizing the dependent variables. By using these criteria, two desirable solutions were obtained, with one having the highest desirability (0.66), which is 52.31% *Ogi* powder and 43.58% termite flour (Table 7). Hence, the consumption of the supplemented *Ogi* powder from the blends of 52.31% *Ogi* powder and 43.58% termite flour may limit the risk of macro- and micro-nutrient deficiencies associated with the consumption of complementary foods in developing countries.

**Table 7:** Criteria for optimization of the chemical and phytochemical composition of supplemented *Ogi* powder from the blends of *Ogi* powder and termite flour, and solutions.

Constraints	Goal	Lower limit	Upper limit	Importance	Solutions	
					1	2
*Ogi* powder	Is in range	30.00	110.00	3	52.31	110
Termite flour	Is in range	1.70	58.00	3	43.58	19.3
Moisture content	Minimize	8.59	12.07	3	9.66	10.42
Fat content	Maximize	2.12	9.63	3	7.30	3.06
Protein content	Maximize	6.83	22.23	3	20.26	11.17
Ash content	Maximize	0.84	3.66	3	3.39	2.04
Crude fiber content	Maximize	1.97	4.02	3	3.40	2.21
Total carbohydrate	Maximize	50.21	77.81	3	54.90	69.42
Energy value	Maximize	342.03	376.44	3	370.20	354.43
Iron content	Maximize	1.68	2.43	3	2.20	1.79
Zinc content	Maximize	2.89	3.21	3	3.17	2.99
Phytate content	Is in range	0.03	0.20	3	0.06	0.11
Oxalate content	Is in range	0.02	0.11	3	0.02	0.10
Saponin content	Is in range	0.05	1.03	3	0.24	0.66
Tannin content	Is in range	0.01	0.05	3	0.02	0.03
Desirability					0.66	0.28

## Conclusion

The protein, crude fiber, ash, iron, and oxalate contents increased when the TF was added to the OP, but the amounts of carbohydrates, zinc, phytate, and saponin decreased. As a result, although adding TF to the OP increases its nutritional content, it also reduces some phytochemical elements like phytate and saponin. However, supplemented *Ogi* powder of higher protein, ash, and iron contents than those of the control sample could be achieved by from SO9, SO3, and SO8. But to produce a termite flour supplemented *Ogi* powder, which may contribute to the nutritional and phytochemical constituents, the response values of fat content, protein content, ash content, crude fiber, carbohydrate, energy value, iron, and zinc contents were maximally optimized while minimizing the moisture content. Also, phytate and saponin contents were kept within range as the phytochemicals. The *Ogi* powder and termite flour were also kept within range as the independent variables while optimizing the dependent variables. By using these criteria, two desirable solutions were obtained, with one having the highest desirability (0.66), which is 52.31% *Ogi* powder and 43.58% termite flour. Therefore, blending 52.31% *Ogi* powder and 43.58% termite flour could produce a supplemented *Ogi* powder with nutritional and phytochemical constituents than those of the control sample. While the supplemented *Ogi* powder could help lower the rate of protein-energy malnutrition, its amino acid, mineral, and β-carotene profiles need to be assessed.

## Data availability statement

The original contributions presented in the study are included in the article/Supplementary material, further inquiries can be directed to the corresponding authors.

## Author contributions

WA: Conceptualization, Data curation, Formal analysis, Investigation, Methodology, Software, Supervision, Validation, Visualization, Writing – original draft, Writing – review & editing. FRF: Conceptualization, Data curation, Formal analysis, Investigation, Methodology, Validation, Visualization, Writing – original draft, Writing – review & editing. BM-D: Conceptualization, Data curation, Funding acquisition, Investigation, Methodology, Project administration, Resources, Supervision, Writing – original draft, Writing – review & editing.
