# Supplementary figures and images for: Influence of adding edible termite flour to Ogi powder: its chemical and phytochemical composition
Source: Front Nutr. 2024 Jul 5;11:1403660. doi: 10.3389/fnut.2024.1403660 (PMC11258035; doi:10.3389/fnut.2024.1403660)

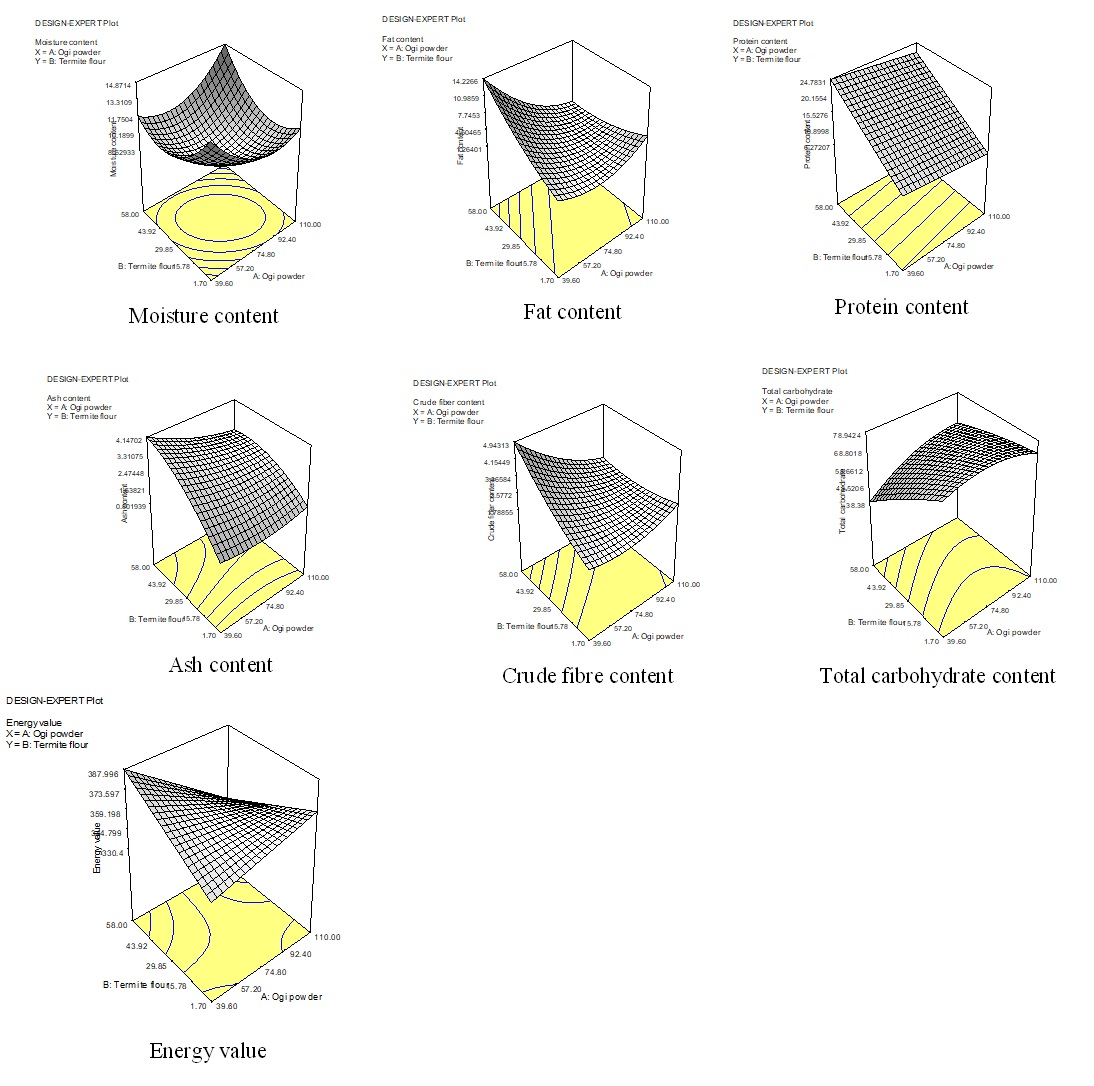

Supplement: Supplementary file 1 [file Image_1.JPEG]

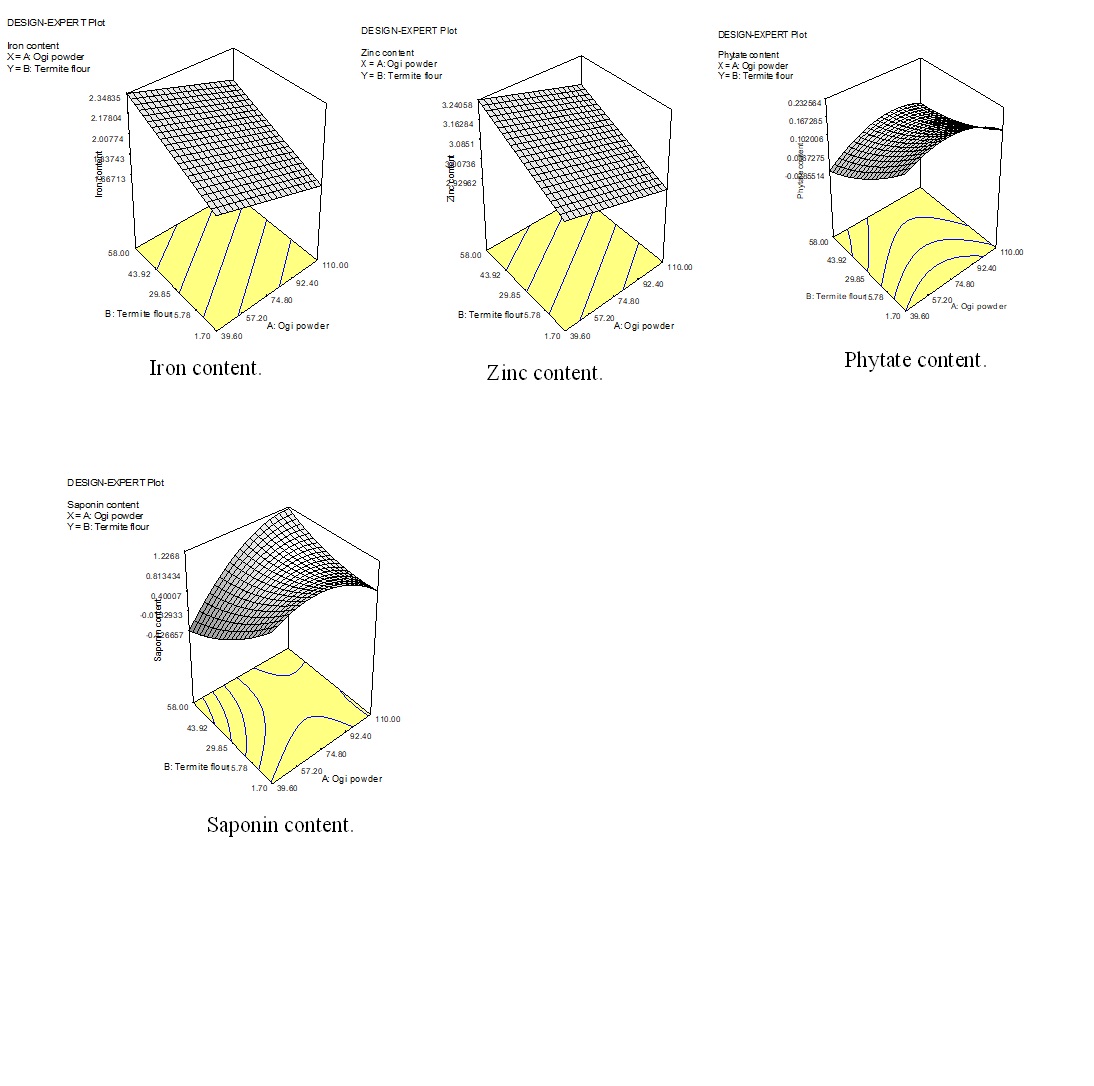

Supplement: Supplementary file 2 [file Image_2.JPEG]
